# Improving mental health care transitions through information capture during admission to inpatient mental health services: a quality improvement study

**DOI:** 10.1186/s12913-021-07136-2

**Published:** 2021-10-21

**Authors:** Natasha Tyler, Nicola Wright, Kyriakos Gregoriou, Justin Waring

**Affiliations:** 1 NIHR Greater Manchester Patient Safety Translational Research Centre, University of Manchester, Manchester, United Kingdom; 2 School of Health Sciences, University of Nottingham, Nottingham, United Kingdom; 3 NHS Derbyshire Healthcare Foundation Trust, Derby, United Kingdom; 4 Health Services Management Centre, University of Birmingham, Birmingham, United Kingdom

**Keywords:** Care Transitions, Mental Health, Inpatient, Information Sharing, Quality Improvement, Communication

## Abstract

**Background:**

Many interventions aim to improve the transition from ward to community at the time of discharge, with varying success. Guidelines suggest that discharge planning should begin at admission, but in reality this is ideal rather than standard practice. We aimed to develop a novel information capture tool during admission that facilitates and accelerates discharge.

**Methods:**

A quality improvement study to develop, implement and evaluate a novel tool that improves information capture upon admission to acute mental health wards within a single English National Health Service (NHS) trust. We developed the tool by synthesising existing evidence and working with multi-agency and multi-disciplinary professionals in two co-design workshops. During implementation the tool was piloted on three wards. Ethnographic observations (145 h) and interviews (45) were used to evaluate the implementation of the tool across the three wards. Thematic synthesis was used to consolidate the findings.

**Results:**

The tool developed considerably as the process evolved. The finished product is a list of 10 information categories that should be captured from external agencies upon admission to hospital to facilitate discharge planning to community settings. Reported advantages of the tool were: (1) facilitating confidence in junior staff to legitimately question the suitability of a patient for an acute ward (2) collecting and storing essential information in a single accessible place that can be used throughout the care pathway and (3) collecting information from the services/agencies to which patients will eventually be discharged.

**Conclusions:**

Improving the quality of information at admission has the potential to facilitate and accelerate discharge. The novel tool provides a framework for capturing this information that can be incorporated into existing information systems. However, the introduction of the tool exacerbated complex, fragile distributed team dynamics, highlighting the importance of sociocultural context in information flow transitional interventions within distributed teams.

**Supplementary Information:**

The online version contains supplementary material available at 10.1186/s12913-021-07136-2.

## Background

At any one time in the UK, 1 in 6 adults will be experiencing a diagnosable mental health condition [1]. The majority will be treated by primary care professionals or IAPT services (Improving Access to Psychological Therapies). However, in 2019-2020 over 54,000 people were admitted to adult inpatient mental health services in England [2]. A Care Quality Commission Report stated that 36 % of NHS mental health trusts were rated as ‘requires improvement to be safe’ [3]. The reasons for inadequate safety on inpatient wards has been attributed to numerous factors, including ineffective information systems, staffing levels, difficulty in accessing services, medications management and physical/environmental issues [3]. A particularly dangerous time in the care pathway is transition, i.e. movement in or out of a care setting at admission, hand-over, transfer or discharge [4]. In a review of English National Health Services (NHS) patient safety incident reports in mental health, almost 10 % were categorised within ‘Access, admission, transfer, discharge’ [5].

Delayed discharge is a particular problem in mental health services. Although there is limited consensus on the definition of ‘delayed discharge’ in this population, research suggests 14 % of UK mental health patients experienced delayed discharge [6], i.e. where they are determined as medically appropriate to leave hospital but are unable to leave due to problems with securing onward care services. Delayed discharge has financial consequences for healthcare organisations, in addition to many human consequences such as stressed, bored and anxious patients, an increase in serious incidents, potential delays in admitting appropriate at risk service users or the premature discharge of others and increased risk of dependence on inpatient care [7].

Interventions to improve safety in mental health care transitions have aimed to reduce readmission [8–10], reduce suicide post-discharge [11, 12] or improve medication management [13, 14]. Few discharge interventions focus explicitly on improving information or knowledge sharing between services, but many acknowledge the importance of communication [9]. Yet, information flow has been described as an important element of patient safety in care transitions from acute to primary care [15, 16]. Research concerning care transitions, primarily hospital discharge, consistently shows that ineffective information flow and co-ordination between health and social care professionals poses a threat to quality and safety [4, 16, 17].

The most common threats to timely and efficient hospital discharge are often related to notifying and organising ‘external services’ [18]. One reason for this common problem is that hospital staff may lack important information at discharge to facilitate discharge planning. In particular they miss important information related to the person’s personal circumstances in the community which could easily be recorded or collated at the point of transition into the hospital, thereby enabling discharge planning from the point of admission. Literature suggests that planned rather than ad hoc actions are essential to address suboptimal information sharing in fragmented care settings [19]. In England and Wales, NICE (National Institute for Health and Care Excellence) guidelines advise that discharge planning should begin from admission [20], but in practice this is not always the case. Qualitative work has found discharge from mental health settings to be unplanned and unexpected in many cases, leaving patients feeling vulnerable and without control [21].

 This quality improvement study aims to work with healthcare professionals to develop an information capture tool that (a) enables staff to implement best practice guidelines/policy into practice to enable discharge planning from admission, (b) standardises the information collected from referral services.

## Study methods

### Design

A quality improvement study, consisting of three components:

A) Prototype Development: (1) Evidence Review/synthesis (2) Proforma Development (3) Co-design Workshop 1 (4) Co-design Workshop 2.
B) Implementation: (1) Baseline Observations and Interviews (2) Implementation Observations and Interviews.
C) Post-Implementation: (1) Follow up Interviews (2) Development of Final Iteration of the Tool.

### Study settings

The quality improvement study was carried out within a single English National Health Service (NHS) trust, for the co-design process we worked primarily with three acute adult inpatient mental health wards within a single campus that were in close proximity to one another (however staff from other campuses within the trust attended workshops). All of the wards had 20-22 bed capacity, were almost exclusively full with a 93-95 % occupancy of patients physically in beds and a remaining 5-10 % on leave but still admitted to each ward. This at times resulted in wards having 24-27 patients allocated to their ward with only 20-22 beds available as several patients would be on leave from the ward with no bed to return. Each ward aimed to have 15 qualified nurses and 15 unqualified staff in total, (around 5-7 per day shift) but were frequently understaffed. The wards were understaffed by one member during most of the observations, because of this plans would often change on the day of observation. Admissions to the wards were both informal/voluntary and also under the Mental Health Act 1983/2007. The wards were mixed-gender wards, two wards cared for adults (18 to 65 years) and one was a mixed adult/older adult ward (18 years plus), the latter was the pilot ward.

### Co-design

A great deal of health research frequently addresses questions and outcomes of limited relevance to clinicians, patients and other end-users [22]. Co-design is one method that facilitates patient, clinician and other end-user engagement in health research. However, definitions and approaches to co-design in the current literature is complex, contradictory and poorly synthesised [23]. Some reviews of co-design use conceptual frameworks to classify levels of involvement, for example “(1) *consultation (where researchers seek the views of the public on key aspects of the research);* (2) *collaboration (an on-going partnership between researchers and the public throughout the research process)* [and]; (3) *‘publicly led’ (where the public designs and undertakes the research and where researchers are only invited to participate at the invitation of the public)*” ([24], p. 106). Different approaches to co-design also use different stakeholder groups, for example some work with patients and the public and some with healthcare professionals or end-users of research, or all/a number of groups [23]. We worked in partnership with a single group (healthcare professionals) only and our work fits primarily into category 2: *collaboration (an on-going partnership between researchers and the public throughout the research process), whereby we worked with professionals in a single trust to develop a tool that meets their needs.* Co-design was used throughout this project in distinct workshops, but also implementing changes to the tool based on the professionals experiences and perspectives (via interviews and observations) each time the tool was adapted.

### A) Prototype/tool development

#### 1. Evidence review and synthesis

We examined existing literature on mental health care transitions and found very little evidence of interventions that focus on improving information capture/flow or the inpatient admissions process, however there were a great number of interventions focused on discharge from inpatient services [9]. We therefore, synthesised thematic data from focus groups with 52 uni-professional/service user groups, the focus of the discussions was mental health care transitions and the interplay between admission and discharge, see original paper for details [21].

#### 2. Proforma development

Based on the synthesis of the focus group data and the professional knowledge of the authors, the team developed a prototype proforma/tool to collect key information on admission to inpatient services. The tool captured information such as purpose of admission, caring responsibilities and accommodation.

#### 3. Co-design workshop 1

The purpose of this co-design event was to present an initial prototype information capture tool, to ignite discussion and suggestions, we also aimed to map out the existing information/patient flow process. Professionals that were invited to the event all worked within inpatient services and ranged in experience and roles including healthcare assistants, nurses, doctors, bed managers. Twenty-three healthcare professionals of various cadres attended workshop 1 (supplementary file 1).

 The research team presented the prototype tool as a starting point for discussion to professionals who worked in groups of 3-6 participants. Participants were first of all asked to discuss whether this would be useful and feasible within their workplace, and whether this is something they would like to see implemented. Afterwards participants were given the opportunity to critique the tool, suggest additions or removals of information categories or suggest replacements. Each small group were asked to feedback their opinions to the wider group and all information was recorded and collated by the researchers. The research team then met to adapt the tool based on the feedback from the event.

#### 4. Co-design workshop 2

This workshop happened one month after workshop 1. The purpose of this workshop was to present the adapted tool to a smaller group of healthcare professionals, to ensure the changes made as a result of workshop 1 were agreed and representative of the group’s opinions. Nine healthcare professionals attended workshop 2, around half of whom attended workshop 1 (see supplementary file 2). This group were directly involved in the gatekeeping/admission process (primarily lead nurses/bleepholders from the ward and crisis team), This event involved presenting each participant with a printed version of the tool and prompting each participant to discuss the feasibility of implementing that tool within their workplace, suggesting further adaptations, additions or removals of information domains.

### B Implementation

#### Ethnographic style observations and interviews

The tool developed during Part A was implemented on three wards and fidelity and feasibility were qualitatively analysed (see results for description of developed tool). An implementation study was carried out over two months informed by the principles of ethnography [25], in that it aimed to observe first-hand and understand through critical interpretation how the tool was experienced and enacted in everyday clinical practice and in the context of prevailing organisational cultures, routines and structures. Ethnography draws on multiple sources of data, in this case (a) observations, and (b) interviews.

#### Observations

One researcher (NT) conducted 145 h of observations of clinical practice. Observations focused only on professional activities (i.e. bed management, meetings between ward staff, phone calls with external agencies, handovers, multi-disciplinary meetings etc.) No patients were directly observed, nor were staff interactions with patients. The fieldwork strategy aimed to progressively deepen understanding of processes, systems and tools concerning mental health care transitions beginning with 1) baseline observation (understand existing processes of admission by shadowing key healthcare professionals that are involved in processing admissions; followed by 2) implementation observation (observing practice with the tool).

Baseline observations aimed to understand existing tools, processes and systems used for care transitions and the introduction of the new admission tool. Usual practice was observed in the baseline observations (70 h) before the tool was introduced. Implementation was then observed during the first two weeks of using the new tool (75 h). Observations and interpretations were recorded in hand-written journals with on-going summary reports typed up and shared with the wider team to inform ongoing reflections and analysis.

#### Interviews

One researcher (NT) used interviews to concurrently deepen understanding of the tool and implementation by speaking to staff directly about what was observed. Interviews enabled the researcher to ask about existing processes (baseline observations) and the new tool (implementation observations), see supplementary file 3 for interview topic guide.

Forty-Five semi-structured interviews were conducted with 40 unique individuals during the observation period (5 baseline, 36 implementation, 4 follow ups), see supplementary file 4.

A purposive sampling strategy was used. Participants were purposively identified on the basis of observed involvement in care transitions, and were usually recruited to interview whilst the researcher was carrying out ethnographic observations, or through working with service leaders to identify relevant individuals. Whilst most of the interviews were with acute ward staff, we also interviewed staff from associated agencies that were involved in care transitions and were based in close proximity to the acute ward (same corridor) for example, crisis team nurses, bed management team, liaison nurses and housing officers.

 Interviews were semi-structured to understand professional perspectives of mental health care transitions with a primary focus on admission and discharge processes, the interplay between the two and the tool development process. Interviews ranged in length from 10 to 90 min, the majority lasted between 20 and 40 min as they happened only when staff were available within working hours. All interview participants gave written consent ahead of the interview and all interviews were recorded and transcribed verbatim. Participants were interviewed until saturation was accounted for.

 The quality improvement study received favourable approval from Research and Development department at the trust. Information sheets and briefings were provided before each interview and at the beginning of the observations. In advance of carrying out observations of any staff member, either through shadowing, staff meetings or gatherings of multiple staff, written consent was sought from all those who were present in the first instance to be observed for the remainder of the study.

### Analysis

The qualitative data collected from the interviews and observations were analysed thematically using qualitative techniques proposed by Ritchie and Spencer [26] During the familiarisation process the researcher (NT) developed a coding framework focusing on benefits of the intervention and barriers to implementation that affect acceptability and fidelity of the tool. This involved coding the individual participant responses and then grouping these together as ‘meaning units’. These grouped units were then assigned consolidated codes, and the similarities and differences between them were compared. Side-by-side comparison of the raw data in the framework enabled us to see differences between the perceptions of the pilot wards professionals using the tool and the other associated teams. A further consolidation process led to the development of overarching themes to explain the data. The initial and majority of the analysis was conducted by NT. The themes and analysis were then discussed within the wider research team for verification purposes (NW, JW, KG).

### C Post implementation

#### 1. Follow-up interviews

Follow-up interviews were conducted 6 months later. This included 4 key informants who were interviewed twice: two nurses who were shadowed frequently and attended the workshops thereby playing a key role in implementation and two service managers who were integrally involved in the project and leading workshops. The four follow-up interviews with key informants were conducted six months after the initial observations to assess the longitudinal impact of the work and clarify our findings.

#### 2. Development of final iteration of the tool

The final version of the tool developed as a result of the process was created by examining implementation study feedback from staff using the tool. The research team discussed the 33 individual suggestions for improvement that were synthesised from the qualitative results of the implementation study observations and interviews. Qualitative data was synthesised into distinct action points and each one was considered in turn in depth by a team of authors (NT, NW, KG). Sixteen of the suggestions were incorporated, thematically these primarily concerned changing wording and combining/condensing similar questions. The suggestions not included tended to involve a structural change that was outside the capacity of this small project such as ‘generating automated referral on admission when no fixed address to accommodation team’ or ‘introduce accompanying training’.

## Results

### A. Prototype/tool development

#### Original information flow processes

**Fig. 1:**
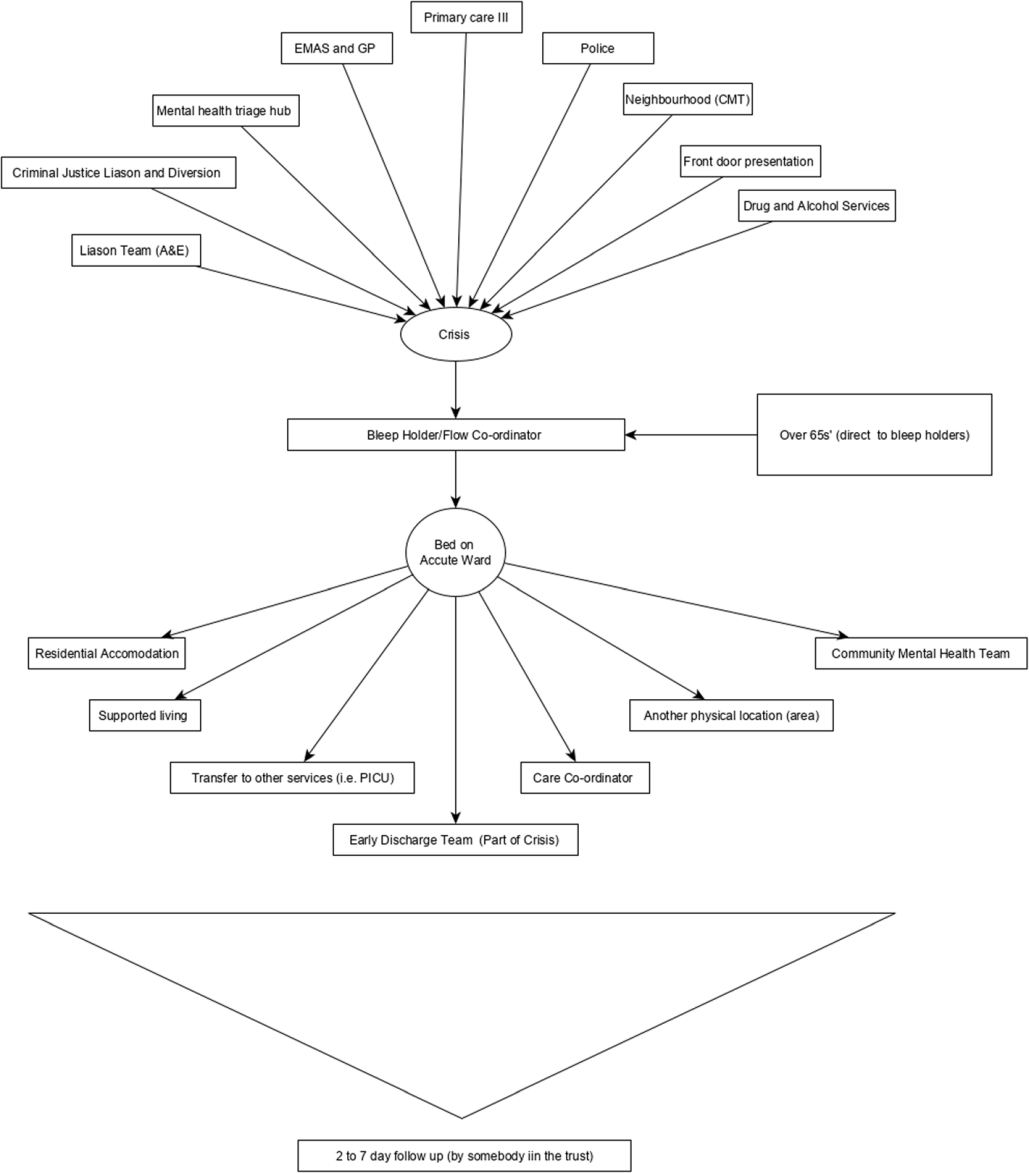
A co-designed flow chart to show the information-flow and patient pathway into the pilot mental health wards

During the workshops, we asked staff to work with us to develop a flow chart to highlight existing information flow from the point of referral to the inpatient ward. Figure 1 shows the referrals originate from another service (i.e. primary care, accident and emergency (A&E) liaison teams, community mental health teams). All staff agreed that referrals go initially to the crisis team (who act as gatekeepers). The crisis team then communicate with the ward via telephone calls to either the bleep holder (usually a ward manager, who is responsible for the bed management of all mental health inpatient wards in the hospital for a particular shift) or a flow co-ordinator (a designated administrator who deals with bed management, however this role existed in one of the trusts other locations, not the pilot wards). It was also noted that the crisis team had a gatekeeping assessment document that captures some of the information in the developed tool. Other personal, clinical and social information that was considered key by stakeholders, would be captured from various sources either during nurse/clinician clerking processes or through existing electronic health records (if known to services).

The field notes from workshop 2 and baseline observations and interviews highlighted information flow and communication during patient admission was rarely straightforward, many individuals informally bypassed the crisis team to communicate directly with the ward (bleep holder).

*‘Community teams would just bypass the crisis team and just go straight to the ward.’* – Head of nursing.

#### Tool development (pre-implementation)

The initial tool underwent numerous changes as a result of the co-design workshops, interviews and observations. In workshop one, the suggested changes were minimal (changes of wording/adding and removing categories) and the discussions were positive in regards to how the tool can improve information flow and delayed discharges. The tool presented in workshop 2, was very similar to that presented in workshop 1, supplementary file 7 shows the changes made.

The discussions in co-design workshop 2 related to how important information capture is as a tool to empower staff to make decisions about safety. In the original systems, crisis teams (who did not work on the wards) controlled who would be admitted and ward managers (bleepholders) felt powerless. It was therefore decided as a result of workshop 2 that the information capturing tool would sit on the ward rather than with the gatekeepers (Crisis team) as it provided an extra level of checking to ensure ward staff have the information they needed, especially as some external agencies may communicate directly with the ward.

‘*Bleep holders physically crying saying they feel disempowered and have no choice but to admit dangerous patients’* – Researcher Field Notes Workshop 2.

The tool was therefore renamed an admissions checklist, see supplementary file 6. It was decided that it would be used to check that the ward professionals have the relevant information needed at admission, rather than become another piece of standard paperwork. The tool consisted of 13 prompts and some key information that would be used during phone calls with the agency referring the new admission to the ward. Supplementary file 5, shows the iteration of the tool that was developed as a result of the co-design workshops and piloted during the implementation stage. We decided to use a paper version of a tool for this brief quality improvement study, as it was easier to implement and adapt when separate from existing electronic health systems.

### B. Implementation

There were three key thematic benefits identified (1) facilitating confidence in junior staff (2) creating a single, accessible documentation and (3) preventing delayed discharge, see Table 1. However, there were several implementation barriers highlighted one overarching theme ‘exacerbation of existing tensions between teams’ comprised of 4 subthemes 1) Poor communication 2) role misunderstanding 3) power imbalances (4) mistrust of other teams. The barriers results in 3 acceptability and feasibility concerns 1) variable buy-in 2) perceptions of tool redundancy 3) confusion regarding tool purpose. The first broad theme concerned the exacerbation of existing tensions between teams and the second theme.

**Table 1:** Key themes grouped in terms of benefits of the tool and implementation barriers

Perceived benefits of the tool	Implementation barriers	Acceptability and feasibility consequences
1) Facilitating confidence in junior staff 2) Single, accessible documentation 3) Reducing delayed discharge	Exacerbation of existing tensions between teams: 1) Poor communication 2) Role misunderstanding 3) Power imbalances 4) Mistrust of other teams	1) Variable ownership and buy-in 2) Perceptions of tool redundancy 3) Confusion regarding tool purpose

#### Key benefits of the tool

There were three broad relative advantages associated with using the tool (1) facilitating confidence in staff to legitimately question the suitability of a patient for an acute ward (2) collecting and storing essential information in a single accessible place that can be used throughout the care pathway and (3) collecting information from the services/agencies that patients will eventually be discharged to that will speed up the discharge process/prevent delayed discharges.

*‘But, I think, from what I’ve seen it’s empowering them…And, I’ve seen a bit of a change in them actually, in terms of stand…you know…sort of, asking the right questions, and challenging, should this person be admitted’* – Acute Service Manager.

*‘this is the information that you’re gathering, that you’re giving to your staff to say, this is the person that’s coming in, these are their risks, these are their needs, this is what we need to help them with, this is the time they’re coming in, this is extra support that they might need. And for me, that’s all part of clerking and introducing that person to the ward’*– Lead Nurse.

Having the information collected as standard and in a single place had’ knock-on’ effects for practice of other staff groups on the acute ward, for example, a junior doctor interviewed was unaware of the tool but had noticed its beneficial implications exemplified in a difference in the quality of information, to which she had access. A similar experience was had by ward staff, who felt that the questions asked using the new tool had a positive effect on the appropriateness of admissions.

*‘This patient got admitted and essentially all that information was there and I’ve not seen the checklist but I’ve seen all of this information on an admission… Yeah, so they may have used it and actually made life a lot simpler’* – Junior Doctor.

It could be that the tool was most beneficial as it collected the information into one place and documentation format that the ward staff needed, were familiar with and involved in the process of design. Having the appropriate key social and clinical information immediately available to ward staff was considered important for reduced delayed discharges. One junior doctor described how all of the information is probably in the online system anyway, but it’s difficult to access and spread across multiple files that may be slow to open. Staff described how delayed discharges could be prevented by enabling earlier discharge planning. Staff had a better understanding of what the proposed purpose of admission was from the perspective of the agency who referred the patient and this enabled appropriate discharge planning.

‘*It was just on [information sharing system] and in different places in the different case notes.’* – Junior Doctor.

*‘I think so, yeah, because you should start looking at it a bit earlier on and you should have a better awareness of why they are admitted, so you should be planning much earlier on. Whereas if you’re not asking these questions, it could be a week before you get that information from the patient. Or, I suppose a day or two before they have seen my medical team’. –* Head of Nursing.

*‘Say it was just someone that the Crisis team wanted to come in for 72 hours, get their medication and they can go, then we can put that that’s what it is, so that that can sort of speed it up then on the admission so the ward staff know that Crisis only want him in for 72 hours-ish.’- Lead Nurse*.

#### Barriers to implementation

**Fig. 2:**
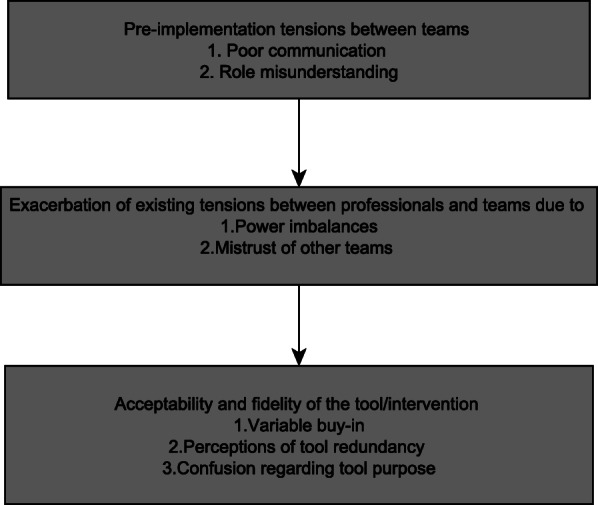
The relationship between thematic results

There were two broad inter-related barriers to implementation, the tool exacerbated existing tensions between teams, primarily (1) poor communication and (2) role misunderstanding by (1) altering power imbalances and (2) increasing mistrust of other teams. This in turn affected the acceptability and fidelity of the new tool in three ways (1) variable buy-in (2) perceptions of tool redundancy and (3) confusion regarding tool purpose. As the results are interrelated (i.e. pre-implementation misunderstanding of other teams roles; which was exacerbated during implementation, resulting in perceptions of tool redundancy) they are not presented as distinct individual themes, Fig. 2 shows the interrelated nature of the themes.

#### Exacerbation of existing tensions

The interviews during the implementation process highlighted poor communication between teams and misunderstanding of roles, associated documentation and capabilities of other teams, in relation to admissions to inpatient wards, this was most prominent between the crisis team and inpatient wards. This was a particular barrier in gaining ‘buy in’ from teams outside of the pilot wards, many of whom felt that the tool was duplicating work or implicitly suggested that they were not performing, further exacerbating tensions. Many of those interviewed from teams outside of the pilot wards (crisis team, bed management team), felt that they were already gathering this information from referral agencies However, interviews with all of the ward staff highlighted that they did not feel they had access to the information they needed to improve safety and patient experience and accelerate discharge.

*‘We gather it all anyway, I make sure that I’ve got it all anyway. I wouldn’t dare ring the bleep holder and say I want a bed and them say, well why and me saying, I don’t know.’*- Crisis Nurse.

*‘I think that is absolute basic stuff that doesn’t get…I think that doesn’t get asked by anybody half the time.’* – Acute Service Manager.

The misunderstanding of roles and documentation of other teams, increased tension during the design process. The bleep holders felt that other teams didn’t understand the pressures they faced trying to secure beds. Although staff from various teams worked together in the workshops, there was a definite tension between ward staff and those from other teams; which was a barrier to implementation and changed the format of the tool after workshop 2.

*‘‘‘Because I’m sure most people think we actually do hide beds up…but people still think we do, so they think they can threaten you or they’ll talk to your manager’ – Lead Nurse*.

*‘In workshop 2, there was evident tension between the crisis team and bleep holders. The crisis team felt that the tool was redundant and a duplication of existing process, whereas the bleep holders felt that they were missing vital information and would like to use the standardised tool to collect this’* – Researcher Field Notes.

#### Acceptability and fidelity

A misunderstanding of the purpose of the tool was evident across the teams during analysis. Whilst the pilot team considered it a tool to only use during certain admissions (i.e. not transfers) external teams considered it a tool to facilitate admission ‘blockings’.

The effect of the tool on power dynamics between teams was profound. Many staff described one benefit as empowerment of more junior staff to be confident in asking questions. However, interviews also suggested that although the tool empowered staff to ask the right questions, it didn’t necessarily give them the power to change anything. An unintended consequence was that some staff reported using the tool as a vehicle to block admissions, by describing the tool as process of rejecting admissions. One interviewee (lead nurse) described the tool metaphorically as a way of depersonalising the rejection process *‘computer says no’*.

*‘Why ask a question, if it doesn’t change anything? …But, I can also see that if you ask for information, and then you challenge it, and you basically get told to pipe down, I can see that you wouldn’t keep asking for stuff’* – Assistant Head of Nursing.

*‘It’s given us the evidence to really robustly challenge that and it’s made the referrers think, when we’ve said, well, have you got a safety plan on? Oh, well, no. Well, I’m not accepting them until we’ve got a safety plan. Because we’ve got the structure of the tool and because we appear to know what we’re talking about, and everybody’s saying the same thing, I think it’s been better’* – Lead Nurse.

The data highlighted that there were inconsistencies amongst professionals on the pilot ward about when the tool should be used. For example, many lead nurses in the pilot wards, felt that it was not necessary to use the tool if there was a transfer back to the ward (for example from an out of area bed or psychiatric intensive care units).

***‘****Yeah, I think admissions that you would use that tool for ’cause you don’t sort of use it for transfers’ –* Lead Nurse.

*‘Most of the calls that have been received today have been for transfers rather than what staff would constitute as ‘new admissions’ therefore the bleep holder today, as well as yesterday, has chosen not to use the tool for transfers for potential mental health act assessments that are not yet definitive admissions. They used scrap paper instead to collect skeletal information.’-* Researcher Field Notes, (day 3 of implementation).

Misunderstanding between teams around the purpose of the tool continued in follow-up interviews. However, follow-up interviews highlighted another beneficial unintended consequence, the work sparked changes in policy around the roles and processes associated with discharge on a local level. However, interviewees highlighted that further work is needed to establish clarity of the tools purpose.

#### Variable ownership and buy-in

There was a lack of knowledge, amongst staff who did not attend the co-design workshops, about the tool being internally developed. Despite all staff being invited to join in the development of the tool at workshops, there was a general consensus, particularly amongst lower-level staff, that they were not involved in the development of the tool and that they did not know who was. This was further exacerbated by lack of communication between those who attended events and non- attending peers.

*‘I don’t know who went to them. Or how often they used it on the other tool since they’ve done the co-production meetings’- Nurse.*

#### Final tool iteration

**Fig. 3:**
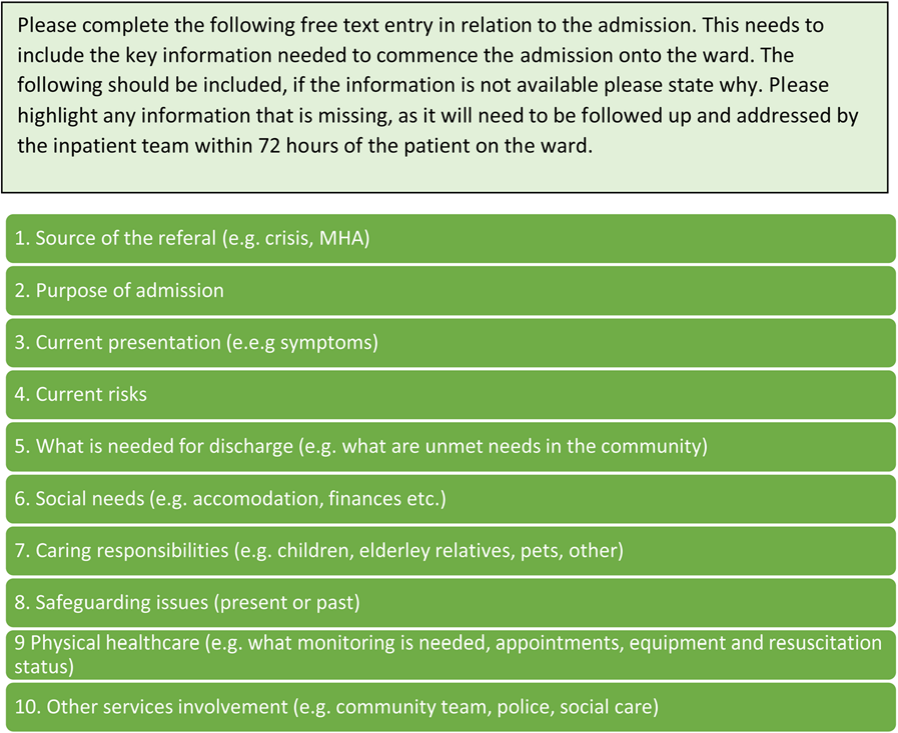
The 10 information categories that were captured the final co-designed tool

As a result of the interview data captured during implementation, 33 potential changes were suggested by staff to improve the tool, see supplementary file 7. The team made final adaptations to the tool based upon this data. The final tool iteration is an information capture pro-forma that can be adapted and used in most existing information systems. The purpose of this tool is to standardise information capture upon admission. The pro-forma enables the healthcare professional that is responsible for liaising with external referral agencies to capture 10 domains of information which participants in this process deemed to be important to enable effective and efficient discharge. The proforma provides prompts and open-text boxes to enable flexibility. The information categories generally concerned personal and social circumstances of patients, see Fig. 3.

## Discussion

The developed tool provides a framework for information capture upon admission to inpatient mental health wards that was developed with healthcare professionals and researchers. It includes ten information categories, including physical health needs, what is needed for discharge, social needs and caring responsibilities. Current interventions in this field, place little focus on information/knowledge sharing and instead focus on preventing outcomes such as suicide, self-harm and readmission [8, 9]. However, knowledge and information sharing is considered a key element of safe care particularly in transitions [15, 16]. Service users and carers consider communication and information sharing a key safety priority [27]. This study highlights how this view is shared by mental healthcare professionals, many of whom feel standardisation of information capture is necessary and beneficial.

On the surface, this work appears to be an introduction of a simple paper-based information capture tool. However, this work addresses a dynamic problem that sits within multiple sets of complex, interacting issues that evolve in an emergent social context. Problems of this kind have been described as ‘Wicked problems’ [28]. Wicked problems are characteristic of complex adaptive systems, such as healthcare systems, furthermore fixes to wicked problems will often contain, within them, other wicked problems [29]. This was evident in this research, whereby a tool developed to capture information became perceived by the users and interacting teams as a tool used to empower a particular team. This work highlights the intricacies of sociotechnical theory for information capture within complex transitional care systems, whereby “… the social requirements of people doing the work with the technical requirements needed to keep the work systems viable with regard to their environments” (p 92). In complex information sharing and communication systems, it is important to consider each subsystem independently and interdependently because optimization of one may have a negative impact on the other [30]. Despite the involvement of multi-professional, multi-agency teams and in the development and implementation of the tool, with consultation at each stage, critical sociotechnical consequences emerged. Information capture at points of transition, has the potential to create novel power tensions between teams.

The follow up interviews (and some of the implementation interviews) suggest the purpose of the tool may have shifted in the stakeholders perceptions from an information capture tool to facilitate timely discharge, to a tool that provides power to a formerly underpowered group to resist admissions. The implementation evaluation suggests that the bleep holders who piloted the tool felt it provided confidence to junior ward staff to question the rationale behind the admission from external agencies. Other staff disciplines and those from other teams sometimes felt it was used as a way of resisting unwanted admissions. The power tension between the bleep holders (who were responsible for utilising the tool) and other gatekeeping agencies (primarily crisis team), who made the majority of decisions about admissions was evident throughout the process. Formatively, it highlights the importance of considering the effect of any information capture tool on power dynamics, as it provides a means of documentation and legitimacy to raised concerns and tensions between groups. In distributed healthcare teams, such as those involved in mental health care transitions, teamwork and communication is a key patient safety issue [31].

This research, like other research into distributed healthcare teams [32], highlights the enormity of the challenge of ‘mutual knowledge’ and ‘shared assumptions’ within distributed teams. The aim of this study was to improve information flow, from a technical perspective (the introduction of a tool) but the sociotechnical environment in which the information capture happened was a distributed healthcare team, with conflicting needs, goals and perspectives. Unlike teams that work together in a single location, there are few structured opportunities for discussions and arguably less mutual knowledge and shared assumptions during transitions of care teamwork [32, 33]. This work highlights how the introduction of information capture tools in care transitions, therefore sit within much more complex, fragile social systems.

Another key formative learning point was in terms of ownership of developed tools, literature suggests that stakeholders are often more receptive to internally developed bottom-up interventions, rather than top-down [34]. Many of the professionals involved in the co design workshop 1 did not work on the three pilot wards and/or were of other professional cadres that they would not use the tool (doctors, ward nurses or healthcare assistants as opposed to bleep holders/lead nurses). There was a lack of communication between those involved in the development process and those involved in the implementation, therefore involving greater numbers of staff from the specific pilot wards or improving dissemination of key learning points from staff workshops could be a crucial way of influencing perceptions of interventions as 'internally developed'.

## Strengths and limitations

The strength of the tool, is that it provides a vehicle to enable information capture at admission that aims to reduce delays in discharge due to social factors. It also provides a way of capturing important social information in a single accessible place.

This study was conducted within a single trust so applicability to other trusts cannot be assumed. However, the key learning points are generalizable to other locations. For example, whilst the bleep holder and crisis team dynamic is probably specific to this location, as it’s based upon localised processes, considering the effects novel information sharing tools have on team dynamics and power dynamics within distributed teams is key. This study was limited as we were unable to involve service users in this work as it was service improvement, so we had to work solely with professionals in the workshops and the implementation study. We did not capture quantitative data, therefore we cannot assess whether there was a direct impact on delayed discharge or length of stay. As this was service improvement study, rather than research, a future larger empirical quantitative analysis of the effect of this tool on length of stay or delayed discharge is now necessary.

Due to the ethnographic nature of data capture, the primary researchers own biases may have affected her interpretation the findings. The benefit of not having experience or expertise in mental health services, arguably prevented professional biases or assumptions, but could also have limited her baseline understanding of complex sociotechnical environment that this research was embedded within. Furthermore, the wider team’s professional assumptions as academics and healthcare professionals may have affected their interpretation of the findings during analysis.

### Future directions

We devised ten key information categories to capture upon admission inpatient wards. Future research is needed to robustly quantitatively assess the effects of implementation of this tool on delayed discharge rates and average length of stay. Future work should look to further test the tool by (a) agreeing with experts whether the 10 items are applicable on a national/international scale, (b) thoroughly assessing how this could fit within existing practice, systems and sociotechnical culture, and (c) robustly, empirically testing the tool (i.e. pilot randomised controlled trial (RCT)).

## Conclusions

The novel tool provides a potential framework for capturing key personal, social and clinical information that can be incorporated into existing information systems. The results suggest that improving the quality of information captured upon admission has the potential to facilitate and accelerate discharge. The study also highlighted the importance of sociocultural context in information flow transitional interventions within distributed teams, whereby the introduction of the tool exacerbated complex, fragile distributed team dynamics.

## Supplementary information

**Additional file 1**

## Data Availability

The datasets generated and/or analysed during the current study are not publicly available as they are qualitative in nature, but are available from the corresponding author on reasonable request.

## Abbreviations

IAPT: Improving Access to Psychological Therapies
NICE: National Institute for Health and Care Excellence
NHS: National Health Service
UK: United Kingdom
