# Supplementary material for: Improving mental health care transitions through information capture during admission to inpatient mental health services: a quality improvement study
Source: BMC Health Serv Res. 2021 Oct 21;21:1132. doi: 10.1186/s12913-021-07136-2 (PMC8529804; doi:10.1186/s12913-021-07136-2)
Supplement: Supplementary file 1 — Additional file 1 [file 12913_2021_7136_MOESM1_ESM.docx]

| Workshop 1 Attendees | | |
| --- | --- | --- |
| Number | Role | Band* |
| 1 | Lead nurse | 6 |
| 2 | Clinical Lead | 7 |
| 3 | Nursing Assistant | 2 |
| 4 | Staff nurse | 5 |
| 5 | Lead nurse | 7 |
| 6 | Lead nurse | 6 |
| 7 | Lead nurse | 6 |
| 8 | Lead nurse | 6 |
| 9 | Staff nurse | 5 |
| 10 | Lead nurse | 6 |
| 11 | Lead nurse | 6 |
| 12 | Lead nurse | 6 |
| 13 | Senior nurse | 7 |
| 14 | Consultant | 8 |
| 15 | Consultant | 8 |
| 16 | Practice Development nurse | 6 |
| 17 | Practice Development nurse | 6 |
| 18 | Senior Nurse | 7 |
| 19 | Lead nurse | 6 |
| 20 | Head of Nursing for Neighbourhoods and Central Services | 8 |
| 21 | Head of nursing | 8 |
| 22 | Assistant Head of nursing | 7 |
| 23 | Staff nurse | 5 |

**Supplementary File 1: Attendees at Workshop 1**

***** The National Health Service (NHS) pays registered nurses under a pay scale system that matches their abilities and responsibilities (banding). A newly qualified nurse starts at band 5.

**Supplementary File 2: Workshop 2 Attendees**

| Workshop 2 attendees |  |  |
| --- | --- | --- |
| Number | Role | Band* |
| 1 | Lead nurse | 6 |
| 2 | Clinical Lead | 7 |
| 3 | Nursing Assistant | 2 |
| 4 | Staff nurse | 5 |
| 5 | Lead nurse | 7 |
| 6 | Lead nurse | 6 |
| 7 | Lead nurse | 6 |
| 8 | Lead nurse | 6 |
| 9 | Staff nurse | 5 |

***** The National Health Service (NHS) pays registered nurses under a pay scale system that matches their abilities and responsibilities (banding). A newly qualified nurse starts at band 5.

**Supplementary File 3: Interview Topic Guide**

**Interview Schedules**

**New Participant**

1. Imagine someone was taking your place at work tomorrow and they don’t know how things work on your ward, how would you explain the admissions process to them?

Prompts :

what are the key stages or decision-points that need to be addressed during this process?

what are the key flows of information and lines of communications you need to progress admission?

what considerations or risk factors do you take into account during the admission process?

How are admissions decisions and processes documented?

1. How have you found the new checklist for admissions?

Prompts:

Does the tool make any difference to processes you highlighted earlier?

Are there any ways that it could be adapted to improve it further?

Which elements are the most useful?

Which elements are the most important?

I there anything you would add or remove?

What would be your opinion on an electronic version?

If I were electronic how would it work?

If you were given the choice would you continue to use this voluntarily in its current form?

1. Thinking about these processes, what things can go wrong or make it complicated?

Prompts:

Are there any particular communication issues or breakdowns – particular people or groups, timing issues, miscommunications?

What impact do these communication issues have on the process?

In what ways is documentation ever missing or incomplete, what implications do these have for admission?

Do you ever find it difficult to complete a thorough admission process, why?

Does the new admissions tool have any effect on this?

1. Thinking about these processes, what things tend to go well and work smoothly?

Prompts:

Are there particular people or groups that help to make the admission process work, if so, how do they contribute?

Does the tool work well when communicating with any particular group? Any groups that it does not work well with?

How does the tool fit alongside existing systems and documentation? Could it be improved?

1. If you can make changes and improvements to the admission process, what would be your top three recommendations?

What would be your top three recommendations in regards to the tool?

1. What is the interplay between admission and discharge, do you have anything to do with the discharge?

Prompts:

What is your opinion of this document following through to discharge?

Would it be possible, is there anything that would make it possible?

How would it work?

**Returning Participant/Second Interview**

1. Could you explain the admissions process using the new checklist?

Prompts:

What are the key stages or decision-points that are addressed during this process?

1. How have you found the new checklist for admissions?

Prompts:

Does the tool make any difference to processes you highlighted earlier?

Are there any ways that it could be adapted to improve it further?

Which elements are the most useful?

Which elements are the most important?

I there anything you would add or remove?

What would be your opinion on an electronic version?

If I were electronic how would it work?

If you were given the choice would you continue to use this voluntarily in its current form?

1. Thinking about these processes, what things can go wrong or make it complicated?

Prompts:

Does the tool have any effect on reducing negative outcomes?

Does the tool improve the process?

1. Thinking about the admission process, what things tend to go well and work smoothly?

Prompts:

Are there particular people or groups that help to make the admission process work, if so, how do they contribute?

Does the tool work well when communicating with any particular group? Any groups that it does not work well with?

How does the tool fit alongside existing systems and documentation? Could it be improved?

1. If you can make changes and improvements to the admission process, what would be your top three recommendations?

What would be your top three recommendations in regards to the tool?

1. What is the interplay between admission and discharge, do you have anything to do with the discharge?

Prompts:

What is your opinion of this document following through to discharge?

Would it be possible, is there anything that would make it possible?

How would it work?

**Supplementary File 4: Table of interview participants in each stage**

| Participant Number | Role | Professional Group | Gender | Participated in Baseline Interview | Participated in Implementation Interview | Participated in 6 month Follow up Interview |
| --- | --- | --- | --- | --- | --- | --- |
| 1 | Bleepholder/Ward Manager | Nurse | F | x | x |  |
| 2 | Bleepholder/Ward Manager | Nurse | M | x |  |  |
| 3 | Bleepholder/Ward Manager | Nurse | F | x |  | x |
| 4 | Crisis Team Manager | Nurse | F | x |  |  |
| 5 | Crisis Team Manager | Nurse | F | x |  |  |
| 6 | Bleepholder/Ward Manager | Nurse | M |  | x |  |
| 7 | Bleepholder/Ward Manager | Nurse | F |  | x |  |
| 8 | Student Nurse | Nurse | F |  | x |  |
| 9 | Student Nurse | Nurse | F |  | x |  |
| 10 | Ward Nurse | Nurse | F |  | x |  |
| 11 | Ward Nurse | Nurse | F |  | x | x |
| 12 | Crisis Nurse | Nurse | M |  | x |  |
| 13 | Crisis Nurse | Nurse | M |  | x |  |
| 14 | Liaison Nurse | Nurse | F |  | x |  |
| 15 | Service Manager | Managerial (non-clinical) | M |  | x |  |
| 16 | Bleepholder/Ward Manager | Nurse | F |  | x |  |
| 17 | Bleepholder/Ward Manager | Nurse | F |  | x |  |
| 18 | Bleepholder/Ward Manager | Nurse | F |  | x |  |
| 19 | Bleepholder/Ward Manager | Nurse | F |  | x |  |
| 20 | Administrator | Administration | F |  | x |  |
| 21 | Administrator | Administration | F |  | x |  |
| 22 | Administrator | Administration | F |  | x |  |
| 23 | Housing Administrator | Administration | F |  | x |  |
| 24 | Junior Doctor | Doctor | F |  | x |  |
| 25 | Doctor | Doctor | M |  | x |  |
| 26 | Hospital Pharmacist | Pharmacy | F |  | x |  |
| 27 | Hospital Pharmacist | Pharmacy | F |  | x |  |
| 28 | Ward Nurse | Nurse | F |  | x |  |
| 29 | Ward Nurse | Nurse | F |  | x |  |
| 30 | Ward Nurse | Nurse | F |  | x |  |
| 31 | Service Manager | Managerial Non-Clinical | F |  | x |  |
| 32 | Clinical Psychologist | Psychologist | M |  | x |  |
| 33 | Head of Nursing | Managerial | M |  | x | x |
| 34 | Assistant Head of Nursing | Managerial | F |  | x | x |
| 35 | Flow Co-ordinator/Bed Manager (elsewhere in trust) | Administration | F |  | x |  |
| 36 | Flow Co-ordinator/Bed Manager (elsewhere in trust) | Administration | M |  | x |  |
| 37 | Bleep holder/Ward Manager | Nurse | F |  | x |  |
| 38 | Bleep holder/Ward Manager | Nurse | F |  | x |  |
| 39 | Care Improvement Lead | Managerial | F |  | x |  |
| 40 | Area Service Manager | Managerial | F |  | x |  |

**Supplementary File 5: First Protocol of Tool (presented at workshop 1)**

**PRE-ADMISSION INFORMATION**

| Name: | **INCL. ANY KNOWN ALIASES** |  | NHS #: |  | | |
| --- | --- | --- | --- | --- | --- | --- |
|  |  |  | EHR No #: |  | | |
| D.O.B: |  |  | Informal? |  | Detained? |  |

| Date & time of phone call: |  |  | Date & time of expected arrival: |  |
| --- | --- | --- | --- | --- |
| Name of person receiving phone call: |  |  | Route of admission: | **i.e. 136, CRISIS, A&E, POLICE, TRANSFER** |

| EHR Referral Completed: | YES  NO |
| --- | --- |
| Accommodation: | **IS THE HOME SECURE NOW?**  **IS THE HOME SAFE / SUITABLE FOR RETURN LATER?**  **IS THE PERSON OF NO FIXED ABODE?** |
| Caring responsibilities:  (e.g., children, elderly relatives, pets, other) | **HAVE THE RELEVANT AGENCIES BEEN INFORMED?**  **IF NOT, WHO WILL INFORM AND WHEN?** |
| Family / carer contact:  (name, address, tel. number) | **IS THIS PERSON THE NEAREST RELATIVE?**  **CAN THEY BE CONTACTED?** |
| Communication needs: | **MAKATON, SIGN LANGUAGE, INTERPRETER?** |

| Admitting person/organisation: |  |
| --- | --- |
| Contact details: |  |

| Previous/current contact with mental health services?  YES  NO | Current mental health care provider:  Previous mental health provider:  Previous admissions:  **WHEN WAS THE MOST RECENT ADMISSION?**  **WHICH WARD / TRUST?** |
| --- | --- |
| Reason for admission on this occasion: | **WHAT IS THE GOAL OF THE ADMISSION (FROM THE ADMITTING TEAM PERSPECTIVE)?**  **WHAT NEEDS TO BE ACHIEVED FOR DISCHARGE TO BE SAFE?** |
| Presenting mental health issues: |  |
| Has capacity been assessed? | YES  NO  If yes, outcome: |
| Presenting physical healthcare issues: | **IF TRANSFERRED FROM A MEDICAL WARD, WHAT INVESTIGATIONS / INTERVENTIONS NEED TO BE CONTINUED / FOLLOWED UP? i.e. IS THE PATIENT MRSA+, BLOOD TESTS TO CHASE, CANNULAS IN SITU? RESUS STATUS? ANY EQUIPMENT NEEDS OR MOBILITY ISSUES?** |

| Advanced Directives: |  | | | | |
| --- | --- | --- | --- | --- | --- |
| Perceived risks:  (to/from self and others) | **IS THERE A PAST HISTORY OF FORENSIC PROBLEMS?**  **IS THERE A HISTORY OF VIOLENT INCIDENTS?**    **ARE THERE ANY RIO ALERTS?**  **IS THE PERSON VULNERABLE?** | | | | |
| Medication:  (mental and physical health meds) |  | When last taken?  (date / time) |  | When next due?  (date / time) |  |
|  |  |  |  |  |  |
|  |  |  |  |  |  |
|  |  |  |  |  |  |
|  |  |  |  |  |  |
| Allergies: |  | | | | |

| GP:  (name / telephone number) |  |
| --- | --- |
| Care Co-Ordinator:  (name / telephone number) |  |
| Date of next CCO meeting / appointment with ward psychiatrist: | **ENCOURAGE THESE MEETINGS TO CONTINUE DURING ADMISSION** |
| Other pending appointments:  (physical healthcare, bail hearings, court cases etc) | **SHOULD THESE BE MAINTAINED DURING ADMISSION?**  **WHO WILL SUPPORT THE SERVICE USER IN THESE MEETINGS?** |

| Other  (e.g. language, cultural, religious needs etc) |  |
| --- | --- |

**Supplementary file 6: Wording of the protocol tool that was implemented on the ward**

**Admissions Checklist**

| Date and Time |  |
| --- | --- |
| Caller Name and Team |  |
| Patient Name |  |
| DOB |  |
| EHR ID Number |  |
| Completed by |  |

1. Is all demographic information up to date?
2. Has crisis completed an up to date gatekeeping assessment?
3. Are risks clearly highlighted and manageable within an acute inpatient ward?
4. Is the admission safe? What have you done about this?
5. Is the purpose of the admission clear with an outline of treatment requirement?
6. What is needed for discharge?
7. What is the estimated date of discharge?
8. Is there a clear explanation of why the person cannot be cared for at home? What is this?
9. Is there an exit strategy
10. What care and support do they have in the community? Please obtain contact details if yes. Are they involved in the admission, if not why?
11. Are there any other none mental health concerns?
12. Do you have any concerns about this admission?

**Supplementary File 7: Changes to the tool implemented at stage.**

**Suggestions Changes from Workshop 1**

| **Suggested Change** | **Action** |
| --- | --- |
| Resus status | Added to physical prompts |
| Past admissions completed | Changed the box (previous admissions) |
| Capacity – has capacity been assessed? | Added into presenting mental health issues |
| Where is the information coming from | To discuss in workshop 2 |
| Advance directives | Added new box by Risk |
| Equipment needs- community, home, what can they bring with them | Added to physical prompts |
| Communication needs | To discuss in workshop 2 |
| Rate of admission | Did not act on – doesn’t seem relevant |
| Accommodation/homeless status | Add to homeless accommodation box |
| What discussion have been had with the gatekeeper | To discuss in workshop 2 |
| What is reasonable for the admission- what needs to be achieved at which point is discharge safe | Added to goal box as prompt |
| Where is discharge back to- cant discharge back to crisis team | To discuss in workshop 2 |
| Have you referred them on EHR | To discuss workshop 2 |
| Safeguarding | added new box |
| Mobility | In physical box |
| Delirium | Exclude as elderly centric |

**Key Journal Notes and Changes from Workshop 2**

- Despite staff receiving the tool well in workshop 1, there was more resistance to the tool in workshop 2. It was a different, smaller group of staff- location and roles.
- Concern over workload of crisis teams and introduction of new initiatives
- Heated discussion between groups about who does what and what’s expected, also what the current admissions information capture procedure and paperwork is
- Crisis team had concerns over duplication and felt they collected this information, ward staff felt they did not already collect this information
- Concerns over staff workload
- Decision was made that the tool would sit with the ward staff and act as a checklist to ensure the information is captured.

**Final 33 Suggested Changes after Implementation Study, Evidence From The Data and Changes Actioned**

| Suggested change | Interview/Observation Data | Changes Actioned |
| --- | --- | --- |
| 1. Change wording to purpose of admission and what is needed for step down | ‘I think sometimes, it’s about firming up that information and asking the direct questions, and what doesn’t often come through from Crisis, is that clear purpose of the admission and the criteria that need to take place to enable them to go back to Crisis. It’s very much, you need to manage this now, but without any clear plan of what we’re supposed to be doing, what our purpose is and what might mean that they can step back to early discharge’ | Yes |
| 2. Getting a structured plan of what acute admission aims to address to allow BH/wards to generate timescales and speed up discharge | ‘What we could really do with, is a structured plan of what we need to address, so’s that we can put some timescales on that and then we can…out of that, would fall out what needs to be met. So once we’ve reviewed medication and got them on an alternative that can then be monitored in community, they could be discharged.’ | Yes |
| 3. Add in ‘unmet needs in the community’ | ‘Well, I’m wondering if we could put something in about unmet needs in the community, or identified…I was going to say needs again, but that’s not right, is it. So there’s got to be clear, identified concerns or issues. So if we were to get those down to formulate, almost like these are the areas of need or concern that we’ve identified, that mean that this person can’t manage at home, we can then turn them into meaningful interventions, from a ward perspective, whether that’s using our OT service or psychologist, or dietitians. Because we’ve got access to all those services, but if all those were ready for when we came in, so it may be, is there a place for other services during inpatients?’ | Yes |
| 4. Capturing important social information that could generate an intervention that will speed up discharge | ‘if somebody’s house is in an absolute state because they’ve not been self-caring, and their diet is terrible, that automatically says, dietitian, it automatically says, look at housing, do we need get the CPC involved to get the house clean, before that person can even think about going home? We can then concentrate on the reasons for the deterioration, the not attending to their ADLs, their lack of an ability to maintain their house. You know, it may be that they can’t budget properly, but you won’t know that until they come in to us. But the housing’s definitely there, as they’re admitted, the ADLs are definitely there, the fact that they’re undernourished is definitely there.’ | Yes |
| 5. This should be part of your inpatient clerking. | ‘Because this is the information that you’re gathering, that you’re giving to your staff to say, this is the person that’s coming in, these are their risks, these are their needs, this is what we need to help them with, this is the time they’re coming in, this is extra support that they might need. And for me, that’s all part of clerking and introducing that person to the ward, and it’s the start of your assessment.’ | No, as this was not intended to be used by Doctors. |
| 6. This needs to be part of the patient’s clinical record | ‘Now I don’t know whether they would be part of those questions that could pull the information through from say, the gatekeeping assessment. If there’s bits we could pull through from the gatekeeping assessment, because there’s a bleep holder, if this is in the gatekeeping assessment and we go onto PARIS and we can read it all, we wouldn’t have to ask these questions. So I think this is where we dovetail with the referrers. So whether it sits on the gatekeeping assessment or a referral, and the referral information then pulls through into a clinical record, rather than lose that information’ | Yes, now able to go into any clinical records |
| 7. If the tool moves to Crisis needs to have a way of wards passing clinical judgement | I would still envisage us and I’m sure the other sort of senior nurses would still envisage us having a clinical type duty person for each shift, who would then be the discussion with Crisis as part of the gatekeeping process.  ‘I think there should still be that safeguard, because they’re sat in their world, in their office downstairs, they’re very sheltered from what we’re doing here, as are we sheltered from what they’ve got on, from a Crisis perspective. So I think there still needs to be that two way discussion with the clinical areas, to say, right, I’ve got this person, they appear to be appropriate for admission. These are the risks, what are you currently managing, is this going to be a problem? And we’d either say, yes, or no. Whatever reason, yes or no. If it’s, no, then we would agree with them, a date and time of admission. If it’s a yes, then obviously that needs discussing further and perhaps escalating, if it’s going to be a problem’ | N/A |
| 8. Remove Estimated Discharge Date – not something referrer should know | I think sometimes the estimated date of discharge might be, you know, it would be a guess...  ‘We’re not heading towards discharge dates at the moment. We’re just not able to do that.’  ‘what’s the anticipated length of stay for this spell? And you can get that from medication changes and things. But if they’re clear about what the purpose of the admission is, they should be clear about what the anticipated length of stay should be. And then that gives us the ability to then start and estimate discharge dates’ | Yes |
| 9. More empty space to replace scraps of paper | ‘So perhaps, yeah, bit more room to do extra notes’ | N/A as no longer a form but a set of information categories to be captured within any system. |
| 10. Separate initial essential information for queries from more detailed information when the admission is progressed | Not that much space, is there, to write on. To have a bit more space, have them as like an essential. So, have loads of blank ones and a shorter one for somebody who wants a bed.    So, before you take, you get in, you ask every single question that’s on there, fill it out and then you say okay, I’ll just look through, I’ve just took all that information, I’ll get back to you.’ | N/A as no longer a form but a set of information categories to be captured within any system. |
| 11. Create an electronic form | ‘on the computer it might be quicker to type, like an online document, ’cause I think they’re trying to go all computerised now.’  Mixed opinions about this, some thought hand written was better | Partially, the categories can now be used in any existing systems going forward. |
| 12. Try and collapse some of the questions into one. | ‘What is this? I mean, that’s in the gatekeeping as well as to why can’t a person be home treated. Is there an exit strategy? Again, that’s the same question as what’s needed for discharge. So, I think asking questions that are the same just irritate people because they feel like you’re duplicating. What care and support do they have in the community? Please obtain contact details. Are they involved in the admission? If not, why’ | Yes |
| 13. Change the name | ‘So, instead of calling it a bleep checklist, call it a bleep bed request handover, does that make sense?... So, then it’s not just on the bleep holder, it’s you working together, it’s your handover’ | Yes |
| 14. Store it on patient records | ‘Yes, to be filed, give to admin, to be download into PARIS and into the patient’s external documents.’  you create a new document like you would with a care…’cause you’re creating new documents, it comes up like safety assessment care plan, everything you need.    So, there should be one saying bed request handover, you click on that and then all the details will already be in there because you’re on that person’s PARIS page and then you just fill it out as you go | Partially, the categories can now be used in any existing systems going forward. |
| 15. Reduce Duplication with Gatekeeping Assessment | ‘a lot of it is already in the gatekeeping. Because what will be frustrating for clicnians is... The gatekeeping document answers quite a lot of this, so they will have gone through this with the Crisis team and then it’ll be a case of then going through it again with the bleep holder. ‘ | Partially, Some categories reduced. Follow up interviews show that the duplication is less problematic now. |
| 16. Ensuring Information is documented in the right place on PARIS | ‘So, a lot of it’s communication, I think. The bleep holders will like it and I think this is all really important stuff, but it should already be being captured and I think some of it is the bleep holders. Even though we’ve cascaded this and said this is what happens, or this is what should happen, they’re not aware of where to find it. So, they’ll sometimes say there’s no gatekeeping documentation on, but they’ve not looked in the right place.’ | N/A |
| 17. Ensuring referrers aren’t being asked twice | ‘Because a lot of it is language as well. You know, people are very sensitive to language and if they feel like they’re being questioned again and again, they get quite frustrated and that’s when people get like... So, say if I’m trying to admit someone and I’m like, right, this person is really poorly and I feel they need to be in hospital. I’ve spoken to the Crisis team and I’ve given them the rationale for why the person needs to come. Then, I’ve spoken to the bleep holder and the bleep holder is like, well, have you done this? I’ve been through all this. ‘ | N/A |
| 18. More clarity in questions, i.e. specifically - is the admission safe? | ‘Is the admission safe? I don’t understand what that means. Is the admission safe? Because it’s things like that. You know, questions that are very vague. If you said to me, is the admission safe, I’d be like, well, obviously if I’ve said the risks are highlighted manageable then it’s a safe admission.’  ‘I wouldn’t say, yes, the risks are manageable and it’s safe for them to come in and then, no, it’s not a safe admission unless that’s asking something else.’ | Yes |
| 19. Be clear about the purpose | Is it an aide-memorie or is it information gathering  ‘Because it seems to have dual purpose because some of those questions are more about... If I’m a bleep holder then this is like an aide-mémoire to make sure I’ve considered everything I need to.’  ‘Where is a bed being allocated? I mean, if you asked me that as someone wanting to refer, I’m going to say I don’t know, that’s your job. And that’s fine for them, but then some of these are things that they need to ask somebody.’ | Yes |
| 20. Collapse some questions into a single question about gateholder assessment | ‘Yes, is all together and it will say, have the Crisis team completed a gatekeeping assessment, and are all these parts been answered? And all the bits of that, that are within this, has this been clearly answered within it? And if not, it’ll be completely within the bleep holder’s right to ask those questions’ | No |
| 21. Change order of items | ‘it’s quite difficult because you’re trying to be on the phone and you’ve got all these questions in a random order at the moment.’ | Yes |
| 22. Automated referral on admission when no fixed address to accommodation team | I suppose you could ask does the person have a permanent home address and if they don’t, then that will be an automatic referral into the homelessness and accommodation or... Because they have social care on the wards as well or both working together to try and address that straightaway. Because that’s one of the main blocks to getting people out, is accommodation. | No |
| 23. Introduce accompanying training | I think it’s definitely training, and I think as part as the admission process, I think better care planning would be a massive boom. So, when the person’s on the ward, if they identify what the person’s need are. So, using the gatekeeping stuff to then identify that in a care plan for somebody. If you’ve done that properly you’ll have identified that the person needs support with starting their medication, but they also need support with finding accommodation and whatever it is. And then kind of actioning that and reviewing it regularly will mean that hopefully it’ll get done | No, but would be good in future developments. |
| 24. Ensuring this information can be used to initiate discharge planning | it’s about if you have the information captured somewhere on admission to start thinking about intervention, what it is that that person needs to become better | Yes |
| 25. This becomes an admission care plan, similar to discharge care plan | So, an admission care plan because they did have discharge care plans in place. If we had an admission care plan, but that formed the basis of the care plan to the person, that would be quite useful | No |
| 26. Use as a checklist for bleepholders to ensure information is right with right people on admission | I think it’d be useful if the bleep holder checklist was about making sure we’re getting the appropriate person with the appropriate information we need and there’d be a next step which would be what has been done with that information? | Yes |
| 27. Remove tool and expand gatekeeping assessment instead | It’s difficult because you could expand the gatekeeping remit a little bit to include other bits of information potentially. | No |
| 28. Combine two items | That’s the one about the exit strategy and what’s needed, which is sort of the same question? | Yes |
| 29. Capture info at admission and send to Drs to plan discharge | If you’ve got all the information already in the whole plans, you can get the ball rolling on the first day. You know, we’ve got a patient currently on our ward where it’s been done in a fairly good way.    We’ve got an exit strategy, we’ve got a planned date of discharge, we know what the patient needs and literally from the moment of admission, I knew exactly what to say to the patient, this is plan, you’re only here for this long, this is what we’re doing. | Yes |
| 30. Explicitly capture rationale for admission | ‘My top one would be if you’re referring someone as an in-patient admission, you give a concrete rationale, so I am admitting this patient because…and there’s like…basically something to put in the background, which is essentially a handover. Like, a breakdown of your handover in a few sentences, this is why I’m referring them, this is just the salient points of it, somewhere that’s easily identifiable, like even if it was to be called rationale for admission. It doesn’t have to be like their whole history, their personal business, | Yes |
| 31. Tool sits with Crisis | I think it still needs to be used by us at the moment. I think it’s something that Crisis should be looking at before they even come to us.  Well, if I could change anything it would be that crisis team 100 per cent manage admissions. | No |
| 32. Check relevance of last question | I suppose the things at the end, are there any other mental health concerns and I suppose that…I’m not sure if it's relevant or not. | Yes |
| 33. Adapt the tool to generate more specific answers to purpose of admission question | Yeah, well, ideally, if somebody had a good, clear purpose of what they wanted from the admission process, but they tend to be couple of answers because in mine you’ll find, improve mental state, because that’s what they always say, and medication review, which are very vague, aren’t they, do you know what I mean, it’s not very specific enough. So I think that’s the issue with it. I think where it might have a benefit is if they crop up again for admission and we could have a quick look at this and say what happened last time and see how, you know, we managed them on the ward and whether they are manageable.  So improve mental health, I mean, in what way, you know, whether they’re engaging more, whether it lessens psychotic symptoms, maybe more succinct to the point of what they want, yeah. And if they’ve done a thorough assessment they should be able to answer that a little bit better than improve mental state | No, this would be covered in any accompanying training going forward. |
